# Personalized embryo transfer guided by rsERT improves pregnancy outcomes in patients with repeated implantation failure

**DOI:** 10.3389/fmed.2024.1369317

**Published:** 2024-05-15

**Authors:** Ning Li, Yisheng Zhang, Rufei Li, Yulin Chen, Lin Huang, Zhuojie Tan, Xiaoying Ban, Ling Zhou, Changlong Xu, Ying Qiu, Rong Li

**Affiliations:** ^1^Reproductive Medical Center of the Second Nanning People's Hospital, Nanning, China; ^2^Reproductive Medical Center of the Guangxi Zhuang Autonomous Region People's Hospital, Nanning, China; ^3^Department of Research and Development, Yikon Genomics (Suzhou) Company Limited, Suzhou, China

**Keywords:** endometrial receptivity, repeated implantation failure, RNA-seq-based endometrial receptivity test, personalized embryo transfer, clinical outcomes

## Abstract

**Introduction:**

Embryo implantation requires synchronous communication between the embryo and maternal endometrium. Inadequate maternal endometrial receptivity is one of the principal causes for embryo implantation failure [especially repeated implantation failure (RIF)] when biopsied good-quality euploid embryos are transferred. An RNA-seq-based endometrial receptivity test (rsERT) was previously established to precisely guide successful embryo implantation. In this study, we aimed to evaluate the effect of personalized embryo transfer (pET) via rsERT on the clinical outcomes in patients with RIF.

**Methods:**

A total of 155 patients with RIF were included in the present retrospective study and were divided into two groups: 60 patients who underwent rsERT and pET (Group rsERT) and 95 patients who underwent standard frozen embryo transfer (FET) without rsERT (Group FET). Reproductive outcomes were compared for patients who underwent rsERT-guided pET and standard FET.

**Results:**

Forty percent (24/60) of the patients who underwent rsERT were receptive, and the remaining 60% (36/60) were non-receptive. The positive human chorionic gonadotropin (β-hCG) rate (56.3% vs. 30.5%, *P* = 0.003) and clinical pregnancy rate (43.8% vs. 24.2%, *P* = 0.017) were significantly higher in Group rsERT patients than in FET group patients. Additionally, Group rsERT patients also showed a higher implantation rate (32.1% vs. 22.1%, *P* = 0.104) and live birth rate (35.4% vs. 21.1%, *P* = 0.064) when compared with FET patients, although without significance. For subpopulation analysis, the positive β-hCG rate, clinical pregnancy rate, implantation rate, and live birth rate of receptive patients were not statistically significant different from those of non-receptive patients.

**Conclusions:**

The rsERT can significantly improve the pregnancy outcomes of RIF patients, indicating the clinical potential of rsERT-guided pET.

## Introduction

Notwithstanding tremendous improvement in *in vitro* fertilization (IVF) for decades, recurrent implantation failure (RIF) is still a challenging and poorly defined clinical phenomenon affecting 5%−10% patients undergoing IVF worldwide (1, 2). To date, RIF has no universally accepted definition, considering the number of unsuccessful IVF cycles, the number of embryos transferred, the quality of embryos, embryo stage, and maternal age (1, 3, 4). According to the preimplantation genetic diagnosis consortium of the European Society of Human Reproduction and Embryology (ESHRE) guideline, RIF was defined as failure to become clinically pregnant when more than three good-quality embryo transfers or ten embryo transfers in multiple transfer cycles are performed (5). Another widely used definition of RIF is the failure of clinical pregnancy after at least four good-quality embryo transfers in at least three fresh or frozen embryo transfer cycles in women under 40 years of age (6).

Human embryo implantation is a highly coordinated and multifactorial process in reproduction, requiring synchronous communication between the embryo and maternal endometrium (7). However, for both patients and doctors, it is frustrating that embryo implantation failure still happens in approximately half of embryo transfers (8–10). Research studies have shown that approximately two-thirds of implantation failures were due to inadequate endometrial receptivity (11, 12).

Endometrial receptivity is defined as the ability of the endometrium to support blastocyst implantation. The window of implantation (WOI), the brief duration of receptivity of the endometrium, usually occurs at days 19–24 of a 28-day menstrual cycle (13). However, the WOI is not constant in women (14). Substantial efforts have been carried out for WOI evaluation in recent decades (15–17). Nevertheless, these methods showed limited predictive value due to subjectiveness and lack of accuracy. Endometrial receptivity array (ERA) was developed based on the expression of a panel of 238 genes using endometrial biopsy samples in 2011 (18). They found that ERA was capable of accurate diagnosis of the WOI (18, 19). Several studies have shown that embryo transfer guided by ERA could avail RIF patients (19–21), whereas many research results have not found much benefit (22–24).

New-generation high-throughput RNA-seq is a powerful tool for comprehensive transcriptome research compared to RNA expression microarray. An RNA-seq-based endometrial receptivity test (rsERT) tool based on 175 differentially expressed genes (DEGs) was established in 2021, and it was found that the WOI could be accurately predicted by the rsERT (25). However, more evidence that the rsERT-guided embryo transfers improve clinical outcomes in RIF patients was needed. Hence, this study aimed to evaluate and validate the effect of personalized embryo transfer (pET) guided by the rsERT on the clinical outcomes in RIF patients and provide further clinical evidence for the clinical potential of the rsERT.

## Materials and methods

### Study population

A total of 155 RIF patients attended the Center for Reproductive Medicine of Nanning Second People's Hospital were recruited from May 2019 to May 2022. Among them, 95 patients (Group FET) did not undergo the rsERT and followed the standard frozen embryo transfer (FET) protocol, and 60 patients underwent the rsERT and rsERT-guided embryo transfer (Group rsERT). In our circumstance, we defined RIF as the failure of clinical pregnancy after at least three high-quality embryo transfers in at least three fresh or frozen embryo transfer cycles (5). A good-quality embryo means a day 3 embryo (≥8 cells, uniform cleavage size, fragmentation rate < 10%) or blastocyst (with a grade ≥3BB). All enrolled patients were aged < 40 years having an endometrial thickness ≥7 mm. Patients with any untreated uterine pathologies and/or endometrial lesions, such as endometriosis, adenomyosis, giant uterine fibroids, abundant intratubal effusion, and recurrent uterine effusion, were excluded. Patients with other factors such as body mass index (BMI) ≥30 kg/m^2^, endocrine pathologies, immune system diseases, and known chromosomal abnormalities were also excluded. This study has been reviewed by the Medical Ethics Committee of the Nanning Second People's Hospital (No. Y2022042), and patients signed an informed consent form before undergoing the rsERT and FET.

### Endometrial preparation, endometrial sampling, and processing

All patients underwent natural cycles or hormonal replacement therapy (HRT) cycles to prepare their endometrium. In HRT cycles, estradiol valerate (4–6 mg/day, Progynova, Bayer, Germany) was taken on days 2–3 of the menstrual cycle. When the thickness of the endometrium assessed by vaginal ultrasound was 8 mm or more, 40 mg/d of oral dydrogesterone (Duphaston, Abbott, USA) and an intravaginal progesterone soft capsule (40–60 mg/d Utrogestan, Besins, France) were administered for progesterone conversion (P+0 on the day of progesterone use). On day 5 (P+5), after progesterone conversion, an endometrial biopsy was performed. In natural cycles, urine/blood luteinizing hormone (LH) was monitored continuously from day 10 of the menstrual cycle, and endometrial tissue was taken 7 days after the LH peak (LH+7) or 5 days after ovulation (ovulation+5). A biopsied endometrial tissue larger than 5 mm was transferred to a specimen collection bottle containing a specific preservation solution (XK-039, Yikon Genomics, China) and inverted up and down 10 times to ensure complete immersion of the endometrial tissue in the preservation solution. Specimens were stored at −20°C for further analysis.

### rsERT performed for receptivity measurement

RNA extraction from endometrial tissue, library construction, and RNA sequencing were conducted, and differentially expressed genes (DEGs) in different endometrial receptive phases were identified according to the previous research (25). The WOI of each biopsied sample was predicted using the significant 175 biomarker genes coupled with artificial intelligence algorithms (25). Therefore, the endometrium can be classified into receptive and non-receptive (including pre- and post-receptive) phases and shows the recommended transplantation time (specific to the hour).

### Embryo transfer guided by the rsERT and outcome measures

For Group rsERT patients, personalized embryo transfer (pET) was performed at the timing of the optimal WOI predicted by the rsERT when blastocysts were transferred and 2 days earlier accordingly when day-3 cleavage-stage embryos were transferred. Moreover, Group FET patients underwent conventional ET directly. All embryo transfers were conducted by experienced physicians under transabdominal ultrasonography guidance.

For Group rsERT patients, finally the clinical outcomes of 48 patients were recorded and analyzed due to the other 12 patients being lost to follow-up. The primary outcomes were positive human chorionic gonadotropin (β-hCG) rate and clinical pregnancy rate. The secondary outcomes were live birth rate and implantation rate. Positive β-hCG was defined as an β-hCG level >10 mIU/mL on day 14 after embryo transfer. Clinical pregnancy was defined as the confirmation of an intrauterine gestational sac with a fetal heartbeat on ultrasound. Live birth refers to deliveries that resulted in live birth. Implantation refers to the observed gestational sacs by ultrasound on day 28 after embryos transfer, with a single ET gestation sac counting as 1 only.

### Statistical analysis

Software SPSS 26.0 was used for statistical analysis in the present study. Continuous variables followed a normal distribution and were represented as mean ± standard deviation. A *t*-test was used for independent samples to compare sample means between groups. The discontinuous variables were presented as composition ratios or percentages (%), and intergroup analysis was performed using the χ^2^ test. Multifactor logistic regression was further performed. A *p*-value of < 0.05 indicated statistical significance.

## Results

### rsERT results in RIF patients

A total of 60 RIF patients underwent rsERT. According to the rsERT results, 24 patients were receptive and 36 were non-receptive (all in the pre-receptive phase) (Table 1). In the receptive patients, the patient's age and the time delay between the proposed transplantation and the conventional transplantation were significantly lower than those in the non-receptive patients (33.5 ± 3.3 vs. 35.7 ± 4.0, *p* = 0.032; 9.1 ± 7.1 vs. 29.9 ± 7.8, *p* = 0.000) (Table 1). Logistic regression analysis showed that age was a risk factor for endometrial receptivity, suggesting that advanced age was correlated with increased risk of impaired endometrial receptivity (OR = 1.165, 95% CI = 1.008–1.347, *p* = 0.038).

**Table 1:** RIF patients underwent the rsERT.

**Characteristic**	**Receptive patients (*n =* 24)**	**Non-receptive patients (*n =* 36)**	***p* value**
Age (years, mean ± SD)	33.5 ± 3.3	35.7 ± 4.0	0.032
Recommended transplantation time delayed from conventional transplantation time (hours, mean ± SD)	9.1 ± 7.1	29.9 ± 7.8	0.000

### rsERT-guided pET improved clinical outcomes in RIF patients

For patients who underwent the rsERT, the pET was performed at the time of the optimal WOI predicted by the rsERT and was accurate to the hour. Finally, the clinical outcomes of 48 patients were recorded due to the other 12 patients being lost to follow-up. The baseline characteristics of Group rsERT and Group FET patients are listed in Table 2. Group rsERT patients had significantly more previously failed embryo transfer cycles than Group FET patients (3.9 ± 1.2 vs. 3.4 ± 0.8, *p* = 0.014) (Table 2). Other characteristics such as age, BMI, number of embryos transferred in the current cycle, endometrial thickness, ratio of the endometrial preparation protocol, and ratio of embryo types transferred were not statistically different between the two groups (Table 2).

**Table 2:** Baseline characteristics and clinical outcomes of group rsERT and group FET patients.

**Characteristic**	**Group rsERT (*n =* 48)**	**Group FET (*n =* 95)**	***p* value**
Age (years, mean ± SD)	35.4 ± 3.4	35.3 ± 3.8	0.850
Body mass index (kg.m^−2^, mean ± SD)	21.2 ± 2.9	21.1 ± 2.6	0.788
Number of previous transplant cycles (pcs, mean ± SD)	3.9 ± 1.2	3.4 ± 0.8	0.014
Number of embryos transferred in this cycle (pcs, mean ± SD)	1.6 ± 0.5	1.6 ± 0.5	0.520
Thickness of endothelium in this cycle (mm, mean ± SD)	9.5 ± 1.5	9.3 ± 1.7	0.456
Type of infertility (%, *n*)			0.030
Primary infertility	47.9% (23/48)	29.5% (28/95)	
Secondary infertility	52.1% (25/48)	70.5% (67/95)	
Endothelial preparation program (%, *n*)			0.907
Natural cycle	20.8% (10/48)	20.0% (19/95)	
Hormone replacement hangindent8pt cycle	79.2% (38/48)	80.0% (76/95)	
Type of embryos transferred (%, *n*)			0.437
Cleavage embryos	33.3% (16/48)	40.0% (38/95)	
Blastocysts	66.7% (32/48)	60.0% (57/95)	
Clinical outcome (%, *n*)			
β-hCG positivity rate	56.3% (27/48)	30.5% (29/95)	0.003
Clinical pregnancy hangindent8pt rate	43.8% (21/48)	24.2% (23/95)	0.017
Live birth rate	35.4% (17/48)	21.1% (20/95)	0.064
Implantation rate	32.1% (25/78)	22.1% (33/149)	0.104

Clinical outcome data were collected and analyzed for patients in the rsERT and FET groups. Group rsERT patients [56.3% (27/48)] demonstrated positive β-hCG, and 43.8% (21/48) patients achieved clinical pregnancies. For Group FET patients, 30.5% (29/95) had positive β-hCG and 24.2% (24/95) were clinically pregnant. These results showed that Group rsERT patients, who underwent pET at the time of the optimal WOI, obtained a significantly higher positive β-hCG rate and clinical pregnancy rate than those from Group FET patients (56.3% vs. 30.5%, *P* = 0.003; 43.8% vs. 24.2%, *P* = 0.017) (Table 2). For Group rsERT patients, 78 embryos were transferred and 25 (32.1%) were successfully implanted. The implantation rate was higher than that in Group FET patients, although without significance (31.1% vs. 22.1%, *P* = 0.104) (Table 2). The live birth rate of Group rsERT patients also exceeded that of Group FET patients, despite the difference not being statistically significant (35.4% vs. 22.1%, *P* = 0.064) (Table 2).

In Group rsERT patients, 39.6% (19/48) were receptive and 60.4% (29/48) were non-receptive. The baseline characteristics of receptive and non-receptive patients are listed in Table 3. Other than significantly less age of receptive patients, BMI, number of embryos transferred in the current cycle, endometrial thickness, ratio of the endometrial preparation protocol, and ratio of embryo types transferred were comparable to those of non-receptive patients (Table 3). As for clinical outcomes, the positive β-hCG rate, clinical pregnancy rate, implantation rate, and live birth rate from receptive patients were not statistically significant different from those of non-receptive patients after the embryo transfer timing was adjusted according to the rsERT recommended optimal WOI (Table 3).

**Table 3:** Baseline characteristics and clinical outcomes of receptive and non-receptive patients.

**Characteristic**	**Receptive patients (*n =* 19)**	**Non-receptive patients (*n =* 29)**	***p* value**
Age (years, mean ± SD)	34.2 ± 3.1	36.2 ± 3.4	0.043
Body mass index (kg.m-2, mean ± SD)	21.1 ± 3.0	21.3 ± 2.8	0.840
Number of previous transplant cycles (pcs, mean ± SD)	4.1 ± 1.0	3.8 ± 1.3	0.531
Number of embryos transferred in this cycle (pcs, mean ± SD)	1.7 ± 0.5	1.6 ± 0.5	0.193
Thickness of the endothelium in this cycle (mm, mean ± SD)	10.0 ± 1.6	9.2 ± 1.3	0.086
Type of infertility (%, *n*)			0.263
Primary infertility	57.9% (11/19)	41.4% (12/29)	
Secondary infertility	42.1% (8/19)	58.6% (17/29)	
Endothelial preparation program (%, *n*)			0.073
Natural cycle	31.6% (6/19)	13.8% (4/29)	
Hormone replacement hangindent8pt cycle	68.4% (13/19)	86.2% (25/29)	
Type of embryos transferred (%, *n*)			0.676
Cleavage embryos	36.8% (7/19)	31.0% (9/29)	
Blastocysts	63.2% (12/19)	69.0% (20/29)	
Clinical outcome (%, *n*)			
β-hCG positivity rate	52.6% (10/19)	58.6% (17/29)	0.683
Clinical pregnancy hangindent8pt rate	42.9% (8/19)	44.8% (13/29)	0.853
Live birth rate	42.9% (8/19)	31.0% (9/29)	0.433
Implantation rate	24.2% (8/33)	37.8% (17/45)	0.206

Furthermore, we evaluated the clinical outcomes from different embryo transfer type. In the rsERT patients, 33.3% (16/48) were transferred cleavage embryos and 66.7% (32/48) were transferred blastocysts. For the FET patients, 40% (38/95) were transferred cleavage embryos and 60% (57/95) were transferred blastocysts. The frequencies of transferred cleavage embryos and transferred blastocysts in rsERT patients were not statistically significant different from those of FET patients. Among Group rsERT patients, we found that patients who underwent blastocyst transfer achieved higher rates of positive β-hCG, clinical pregnancy, live birth, and implantation (62.5% vs. 43.8%, 50.0% vs. 31.3%, 40.6% vs. 25.0%, and 41.7% vs. 16.7%, respectively) (Table 4). The same circumstances also occurred in Group FET patients (43.9% vs. 10.5%, 35.1% vs. 7.9%, 31.6% vs. 5.3%, and 35.0% vs. 7.2%) (Table 4). For patients who underwent cleavage embryo transfers, rsERT patients showed a significant higher rate of positive β-hCG (43.8% vs. 10.5%, *P* = 0.016), while the rates of clinical pregnancy, live birth, and implantation were without statistically significant differences (Table 4). For patients who underwent blastocyst transfer, rsERT patients also showed a significant higher rate of live birth (40.6% vs. 31.6%, *P* = 0.039), while a statistically significant higher positive β-hCG rate, clinical pregnancy rate, and implantation rate were not observed (Table 4).

**Table 4:** Clinical outcomes of cleavage embryo transfer and blastocyst transfer.

**Characteristic**	**Cleavage embryo transfer**			**Blastocyst transfer**		
	**Group rsERT (*****n** =* **16)**	**Group FET (*****n** =* **38)**	***p*** **value**	**Group rsERT (*****n** =* **32)**	**Group FET (*****n** =* **57)**	***p*** **value**
Age (years)	35.5 ± 4.0	36.9 ± 2.7	0.134	35.4 ± 3.1	34.2 ± 4.0	0.157
Body mass index (kg.m^2^)	21.0 ± 2.0	20.9 ± 2.9	0.897	21.4 ± 3.2	21.3 ± 2.3	0.868
Number of previous transplant cycles (pcs)	4.0 ± 1.0	3.3 ± 0.6	0.004	3.9 ± 1.3	3.5 ± 0.9	0.160
Number of embryos transferred in this cycle (pcs)	1.9 ± 0.3	1.8 ± 0.4	0.602	1.5 ± 0.5	1.4 ± 0.5	0.384
Thickness of endothelium in this cycle (mm)	9.4 ± 1.6	9.1 ± 1.4	0.458	9.5 ± 1.4	9.4 ± 1.8	0.744
Endothelial preparation program (%)			0.833			0.715
Natural cycle	31.3% (5/16)	34.2% (13/38)		15.6% (5/32)	10.5% (6/57)	
Hormone replacement cycle	68.8% (11/16)	65.8% (25/38)		84.4% (27/32)	89.5% (51/57)	
Clinical outcome (%, *n*)						
β-hCG positivity rate	43.8% (7/16)	10.5% (4/38)	0.016	62.5% (20/32)	43.9% (25/57)	0.091
Clinical pregnancy rate	31.3% (5/16)	7.9% (3/38)	0.074	50.0% (16/32)	35.1% (20/57)	0.169
Live birth rate	25.0% % (4/16)	5.3% (2/38)	0.102	40.6% (13/32)	31.6% (18/57)	0.039
Implantation rate	16.7% (5/30)	7.2% (5/69)	0.286	41.7% (20/48)	35.0% (28/80)	0.451

### Logistic regression analysis determined risk factors affecting clinical outcomes in RIF patients

A multifactorial logistic regression analysis was conducted to identify factors affecting the rate of β-hCG positivity in patients with RIF. Factors included in the analysis were rsERT (rsERT-guided pET or FET without rsERT), age, body mass index, type of infertility (primary or secondary infertility), endometrial thickness in the current cycle, endometrial preparation protocol (natural cycle or hormone replacement cycle), number of embryos transferred in the current cycle, type of embryos transferred (cleavage embryos or blastocysts), and number of previous embryo transfer cycles. As listed in Table 5, we found that rsERT-guided pET could significantly improve the rate of β-hCG positivity in RIF patients (OR = 3.049, 95% CI = 1.241–7.488, *P* = 0.015). More embryos transferred in the current cycle and blastocysts transferred were also associated with increasing β-hCG positivity (OR = 8.322, 95% CI = 3.040–22.780, *P* = 0.000; OR = 9.547, 95% CI = 3.259–27.965, *P* = 0.000) (Table 5). Other factors such as age, body mass index, type of infertility, endometrial thickness in the current cycle, endometrial preparation protocol, and number of previous transplant cycles were not statistically significantly associated with a positive β-hCG rate (Table 5).

**Table 5:** Multifactorial logistic regression analysis identified risk factors for clinical outcomes in RIF patients.

**Characteristic**	**Odds ratio**	**95%CI**	***p* value**
rsERT (rsERT guided pET vs. FET without rsERT)	3.049	1.241–7.488	0.015
Age	0.895	0.793–1.010	0.071
Body mass index	1.123	0.952–1.325	0.169
Type of infertility (secondary infertility vs. primary infertility)	0.792	0.317–1.979	0.617
Endometrial thickness in the current cycle	0.825	0.624–1.090	0.176
Endometrial preparation protocol (natural cycle vs. hormone replacement cycle)	1.066	0.360–3.159	0.908
Number of embryos transferred in this cycle	8.322	3.040–22.780	0.000
Type of embryo transferred (blastocysts vs. cleavage embryos)	9.547	3.259–27.965	0.000
Number of previous transplant cycles	1.122	0.734–1.715	0.595

## Discussion

RIF is a challenging research topic in human reproduction because it impacts conception in healthy women (1). A major cause of implantation failures is inadequate/impaired endometrial receptivity, which was known to account for approximately two-thirds of implantation failures (12, 26). In the present study, we conducted a retrospective study including 155 RIF patients to evaluate the effect of rsERT-guided embryo transfer on the clinical outcomes and demonstrated that rsERT-guided pET significantly improved clinical outcomes in RIF patients.

Endometrial receptivity and the characteristics of the WOI have been the focus of research since 1937, and several methodologies have been developed for endometrial receptivity or WOI evaluation in recent decades. Noyes et al. in 1950 initially established criteria for dating the endometrial biopsy according to the histological changes of the endometrium around the time of implantation (27). However, along with the increasing knowledge of the endometrium, research studies have shown that histologic dating of the endometrium could not provide an accurate diagnosis of WOI (15, 28). Transvaginal ultrasound is considered widely used for endometrial receptivity evaluation using ultrasonic markers such as endometrial thickness, endometrial volume, endometrial pattern, endometrial blood flow, and endometrial contractions due to its non-invasiveness. However, associations between clinical pregnancy and these endometrial receptivity markers were supported by low to very low quality of evidence, thus limiting their feasibility as diagnostic tests of endometrial receptivity (29). A morphology marker pinopodes, which are mushroom-like membrane projection in the endometrium during the peri-implantation period, were proposed and used for WOI prediction, and demonstrated favorable outcomes in women (30, 31). Nevertheless, strong controversies still existed regarding the clinical value of pinopodes (31). Overall, these conventional methods showed poor ability for WOI diagnosis or endometrial receptivity status assessment because of the subjectiveness, uncertainty of reproducibility, lack of accuracy, and contradictory clinical value.

Given the increasing deeper understanding in the transcriptomic characterization of the endometrial cycle, up- and downregulated genes deciphering the molecular features of a receptive endometrium and endometrial receptivity were recently identified (32, 33). Thus, different molecular predictive tools were designed and established to evaluate endometrial receptivity status and predict the optimal WOI, aiming to optimize embryo transfer timing (34). Win-Test (window implantation test) was developed in 2009 by Haouzi et al. based on the expression levels of 11 genes determined by qRT-PCR to predict the receptive endometrial status and revealed improved clinical outcomes of RIF patients after pET compared to patients undergoing standard ET (35). However, to date, only limited studies were performed on Win-Test to evaluate its clinical efficacy, thus hampering its clinical applications. In 2011, Díaz-Gimeno et al. reported the endometrial receptivity array (ERA) to evaluate endometrial receptivity using microarray technology to detect 238 differentially expressed genes with significant predictive power (18). Many research studies were conducted to evaluate ERA's clinical effectiveness and obtained controversial evidence. Some of them showed that ERA may be useful for both RIF patients and the general population, while some indicated that ERA may be beneficial mostly for RIF patients (20, 36–38). ER Map/ER Grade were other novel molecular tools based on differentially expressed genes, providing accurate evaluation of endometrial receptivity and significant improvement of clinical outcomes (39, 40). Despite these promising results, scarce evidence was reported until now. Comprehensively, the clinical effectiveness of these molecular predictive tools needed to be further addressed by well-designed prospective studies and/or randomized controlled trials. Moreover, neither of these predictive tools were focused on Chinese women.

The rsERT, the new recently RNA sequencing-based endometrial receptivity test, was initially established in a two-phase study of Chinese population and showed a significant increase in clinical pregnancy rates in a small sample size of RIF patients (25). In the present study, we recruited 155 RIF patients to further evaluate the clinical value of the rsERT. According to rsERT results, 40% (24/60) patients were receptive and 60% (36/60) were with a displaced WOI (all in the pre-receptive phase). The frequency of non-receptive RIF patients was slightly higher but within the range of 25–88.5% reported in previous studies (25, 35, 41–43). In terms of clinical outcomes, the rsERT was shown to significantly improve the positive β-hCG rate and clinical pregnancy rate of patients who underwent pET at the time of the optimal WOI according to the rsERT, compared to those from Group FET patients (56.3% vs. 30.5%, *P* = 0.003; 43.8% vs. 24.2%, *P* = 0.017). The implantation and live birth rate of Group rsERT patients were also higher than those in Group FET patients, although without significance (31.1% vs. 22.1%, *P* = 0.104; 35.4% vs. 22.1%, *P* = 0.064). As for non-receptive patients, after embryo transfer timing was adjusted according to the rsERT-recommended optimal WOI, the positive β-hCG rate, clinical pregnancy rate, implantation rate, and live birth rate were comparable to those of receptive patients. In addition, subgroup analysis showed that regardless of cleavage embryo transfer or blastocyst transfer, rsERT patients achieved higher rates of positive β-hCG, clinical pregnancy, live birth, and implantation. These results suggested that WOI displacement was the main cause of implantation failure in the displaced patients rather than the influence of embryonic factors, and their clinical outcomes were effectively improved by adjusting the embryo transfer time according to rsERT results. Therefore, we could conclude that rsERT-guided pET could significantly improve the clinical outcomes in RIF patients, especially those with WOI displacement.

Moreover, despite in Group rsERT patients or Group FET patients, we found that patients who underwent blastocyst transfer achieved higher rates of positive β-hCG, clinical pregnancy, live birth, and implantation, even more obvious in Group FET patients (Group rsERT, 62.5% vs. 43.8%, 50.0% vs. 31.3%, 40.6% vs. 25.0%, and 41.7% vs. 16.7%; Group FET, 43.9% vs. 10.5%, 35.1% vs. 7.9%, 31.6% vs. 5.3%, and 35.0% vs. 7.2%). These results were consistent with those of previous studies. From a recently reported review involving 32 randomized controlled trials (RCTs) which compared the effectiveness of IVF with blastocyst-stage embryo transfer vs. IVF with cleavage-stage embryo transfer, fresh blastocyst-stage transfer was associated with higher rates of live birth and clinical pregnancy (OR = 1.27, 95% CI = 1.06–1.51; OR = 1.25, 95% CI = 1.12–1.39) (44). Similarly, a retrospective cohort study including women who underwent FETs at either the blastocyst stage (*n* = 118,572) or the cleavage stage (*n* = 117,619) showed that blastocyst FET was associated with higher rates of live birth and clinical pregnancy compared with cleavage-stage FET (OR = 1.49; 95% CI = 1.44–1.54; OR = 1.68; 95% CI = 1.63–1.74) (45). Combining our data, these research studies provided solid evidence that fresh or frozen/thawed blastocyst transfer could be a preferred strategy for RIF patients.

Furthermore, we performed multifactorial logistic regression analysis to determine factors influencing clinical outcomes in RIF patients in our cohort. We found that advanced age, higher BMI, secondary infertility, a much thicker endometrium, natural cycle for endometrial preparation, and more previous failed transplant cycles were associated with an impaired positive β-hCG rate, although without significance, whereas via rsERT-guided pET, more embryos were transferred in the current cycle, and blastocysts transfer was shown to be associated with a higher rate of β-hCG positivity in RIF patients (OR = 3.049, 95%CI = 1.241–7.488, *P* = 0.015; OR = 8.322, 95% CI = 3.040–22.780, *P* = 0.000; OR = 9.547, 95% CI = 3.259–27.965, *P* = 0.000), indicating that rsERT-guided pET, blastocyst transfer, and multiple embryo transfers could significantly improve the clinical outcomes in RIF patients.

The requirement for an endometrial biopsy is one of the inevitable problems in the diagnosis of endometrial receptivity. According to some researchers, endometrial scratching is a procedure that aims to create a controlled injury to the endometrium, can boost the rate of embryo implantation, and improve pregnancy outcomes by altering the endometrium's inflammatory response (46). Nonetheless, a large-scale randomized controlled trial published in 2019 (47) showed that endometrial scratching prior to embryo transfer did not improve reproductive outcomes in patients undergoing IVF. As a result, we think the process of endometrial biopsy sampling might not have an impact on the study's findings. Research studies also showed that the quality of the embryo and the patient's own conditions have a greater influence on the reproductive outcomes of FET than endometrial preparation regimens, such as the natural cycles and HRT cycles (48). Thus, the difference in endometrial preparation regimens in our present study had no bearing on the study's findings, when rsERT group patients were suggested to select HRT cycles in order to precisely determine when to transfer embryos. Our findings showed that the rsERT could enhance reproductive outcomes in RIF patients by evaluating the status of the endometrium to see if it is appropriate for embryo transfer and implantation.

Several limitations should be acknowledged. First, the sample size of this study was small, which may impair the precision of our risk estimates. Thus, future studies with larger sample sizes would be needed to further address and strengthen our findings. Second, this is a single-center and retrospective study, and morphological criteria were used to select embryos for transfer, which may not be devoid of embryonic aneuploidies. Therefore, a future multicenter randomized controlled trial of the rsERT combined with PGT-A was needed. Third, 56.3% and 43.8% patients achieved positive β-hCGs and clinical pregnancies, respectively, and implantation failures were still observed in nearly one-third of RIF patients after rsERT-guided pET, suggesting other causes of implantation failures in addition to impaired endometrial receptivity, like endometrial receptivity, pathological disruption, or embryonic factors. Additionally, our present study solely focused on RIF patients, not including general infertile population; hence, future studies were needed to address whether the rsERT could be beneficial for these women. In addition, considering the invasiveness of endometrial sampling and extra mock cycles to wait for embryo transfer in the next cycle according to the rsERT results, the non-invasive and fast endometrial receptivity test tool is our future research direction for achieving embryo transfer in the same cycle.

## Conclusion

In summary, the rsERT was applied on 60 RIF patients, and it was revealed that 60% were non-receptive. pET was performed under the optimal WOI determined by the rsERT and showed that rsERT-guided pET significantly improved the clinical outcomes of RIF patients, providing evidence support for the clinical effectiveness of the rsERT for endometrial receptivity and WOI assessment.

## Data availability statement

The raw data supporting the conclusions of this article will be made available by the authors, without undue reservation.

## Ethics statement

The studies involving humans were approved by the Second Nanning People's Hospital. The studies were conducted in accordance with the local legislation and institutional requirements. The participants provided their written informed consent to participate in this study. Written informed consent was not obtained from the individual(s) for the publication of any potentially identifiable images or data included in this article because this study is a regression study, and all patients completed treatment. The datas in this study does not involve participants identifiable datas, and the publication of the data will not harm the interests of patients.

## Author contributions

NL: Conceptualization, Data curation, Software, Writing – original draft, Writing – review & editing. YZ: Data curation, Software, Writing – original draft, Writing – review & editing. RuL: Data curation, Software, Writing – original draft, Writing – review & editing. YC: Data curation, Software, Writing – original draft, Writing – review & editing. LH: Investigation, Methodology, Writing – review & editing. ZT: Data curation, Investigation, Methodology, Writing – review & editing. XB: Data curation, Formal analysis, Methodology, Writing – review & editing. LZ: Data curation, Investigation, Methodology, Writing – review & editing. CX: Data curation, Investigation, Methodology, Writing – review & editing. YQ: Data curation, Formal analysis, Investigation, Writing – review & editing. RoL: Conceptualization, Funding acquisition, Resources, Validation, Visualization, Writing – review & editing.
